# Successful surgical treatment of idiopathic tractional corectopia complicated by persistent pupillae membrane. Case report

**DOI:** 10.1016/j.ijscr.2025.111908

**Published:** 2025-09-03

**Authors:** N. Bobrova, O. Dovhan, T. Romanova

**Affiliations:** Pediatric Ophthalmic Pathology Department, SI “The Filatov Institute of Eye Diseases and Tissue Therapy of the NAMS of Ukraine”, 49/51 Frantsuzkyi Bulvar, Odesa, 65015, Ukraine

**Keywords:** Idiopathic tractional corectopia, Discoria, Microcoria, Persistent pupillary membrane, Anterior embryotoxon, Pupilloplasty, Transparency lens preservation

## Abstract

**Introduction:**

Idiopathic tractional corectopia (ITC) is a rare congenital anomaly affecting the pupil's position and shape. In the literature, there are isolated cases of surgical treatment for ITC, which caused visual axis obstruction.

**Case presentation:**

А 1-year-old child complained of low vision in the left eye and was diagnosed with ITC, microcoria, discoria, persistent pupillary membrane (PPM), anterior embryotoxon, and microphthalmos. Surgical removal of PPM with anterior embryotoxin was performed, restoring the pupil's size, position, and shape. Lens transparency was preserved. The anatomical structure of the anterior eye was restored. Postoperatively, the child received local/generalanti-inflammatory therapy and pupil massage. IOP remained normal, and VA improved to 0.13 Teller's cards (0.9 logMAR). At 12 months after surgery, stable optical and cosmetic results were observed with VA increased to 0.2 Teller's cards (0.7 logMAR).

**Clinical discussion:**

ITC is combined with PPM, usually unilateral, and can progress, leading to visual axis obstruction and amblyopia development. Various treatments have been proposed, involving PPM strand dissection and pupil sphincterotomy, which can cause complications such as loss of pupil shape and ability to dilate evenly, resulting in a cosmetic defect. The newly developed surgical technique helps prevent these complications.

**Conclusion:**

The case demonstrates the restoration of pupil position, size, shape, and function in ITC, achieved by atraumatic PPM removal as opposed to sphincterotomy. Preservation of lens transparency and accommodative ability improved VA and prevented amblyopia development. For the first time, surgical anterior embryotoxon removal without complications for the cornea, iris, and anterior chamber angle was described.

## Highlights

• Idiopathic tractional corectopia with persistent pupillary membrane causing vision loss needs early surgical intervention
• Careful PPM removal allows for restoration of normal pupillary anatomy and functions
• There can be a transparent lens under the removed PPM and every effort should be made to maintain its transparency
• Removal of anterior embryotoxon fused to PPM was performed without cutting instruments - only with collet forceps and spatula
• No complications in post-op period after surgical removal of anterior embryotoxon and as described for the first time

## 1 Introduction

Corectopia refers to the displacement of the pupil's position from its central location in the iris. Idiopathic tractional corectopia (ITC) is congenital isolated unilateral anomaly affecting both the position and shape of the pupil. There are isolated reports of clinical cases of ITC in the literature [1,2]. In this paper, we present a case of surgical reconstruction of the pupil in congenital ITC complicated by the presence of a persistent pupillary membrane (PPM), resulting in the restoration of the correct position, shape and size of the pupil, which regained the ability to freely constrict and dilate in all meridians.

The work has been reported in line with the SCARE criteria [3].

## 2 Case presentation

A 1-year-old boy was hospitalized at the Department of Pediatric Ophthalmopathology due to complaints of low vision in his left eye, as reported by his mother.

The child was born to healthy parents. There was no family history of genetic disorders. The pregnancy proceeded without complications. The child was delivered by caesarean section at 35 weeks, weighing 3100 g, and developed normally for his age. There was no allergic history.

Visual acuity was 0.2 Teller's cards (0.7 logMAR) in the right eye and light perception in the left eye. When using mydriatics, the visual acuity of the left eye did not increase.

The child underwent the examination under general anesthesia.

The right eye appeared clinically healthy.

**Fig. 1:**
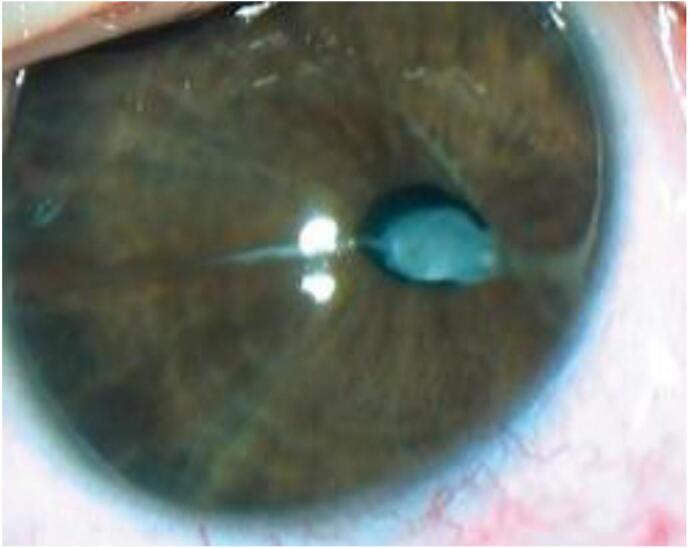
The preoperative appearance of the left eye shows idiopathic tractional corectopia and obliteration of the pupil due to a persistent pupillary-iris-lenticular membrane. The membrane extends as an embryotoxon at 3.30 o'clock and is hidden within the iris thickness as a dense white strand at 9.00 o'clock.

In the left eye, biomicroscopically, the cornea appeared clear, the anterior chamber was of medium depth, the anterior chamber aqueous humor was transparent, and the anterior embryotoxon surrounded the entire circumference of the anterior chamber angle (ACA). The pupil was oval, reduced in size, and displaced nasally at 3.30 o'clock. Within the pupil lumen, there was a PPM in the form of a dense fibrous membrane that covered almost the entire pupillary area, with the exception of a very thin strand measuring up to 0.1 mm wide at the top. The PPM adhered closely to the anterior capsule of the lens and extended to the iris in two strands measuring up to 1 mm in width. Of these, one was hidden in the thickness of the iris at 9 o'clock, while the second, located at 3.30 o'clock, pulled the pupil and iris toward the limbus, where it extended as an anterior embryotoxon (Fig. 1). In medication-induced mydriasis, there was limited dilation of the upper half of the pupil, the PPM remained motionless, and the lens was visualized only above the PPM and appeared transparent. Ophthalmoscopy was challenging to perform due to the presence of PPM. However, a red reflex from the fundus could be seen in the area of pupil dilation. The intraocular pressure was 22.0 and 21.0 mm Hg in the right and left eyes, respectively. Ultrasound biometrics showed 3.3–3.7-20.43 mm and 2.9–3.7-19.7 mm, in the right and left eyes, respectively. Ultrasound scanning of the left eye revealed a sonographic anterior chamber of average depth, measuring 2.8 mm. A strand in the form of anterior synechiae measuring 1.0 mm passed through the area of the pupil in front of the lens. Single medium echogenic masses were detected within the lens. Single-dot-fiber structures of low echogenicity were observed in the vitreous. The retina was adherent. A minor excavation of the optic nerve was detected. Keratometry measurements were 42.75^D^ and 42.5^D^ for the right and left eyes, respectively. Sciascopy revealed Em in the right eye and Hm + 2.0^D^ in the left eye.

The child was diagnosed with idiopathic tractional corectopia, microcoria, discoria, persistent pupillary membrane with iridio-pupillary-lenticular attachment, anterior embryotoxon, and Grade I microphthalmos in the left eye and emmetropia in the right eye.

Given the presence of obscurative PPM, which caused a sharp decrease in visual acuity to light perception, early surgical intervention was advised. This intervention included the removal of PPM and the restoration of the size, position, and shape of the pupil, while attempting to maintain the lens's transparency.

### 2.1 Surgery procedure

**Fig. 2:**
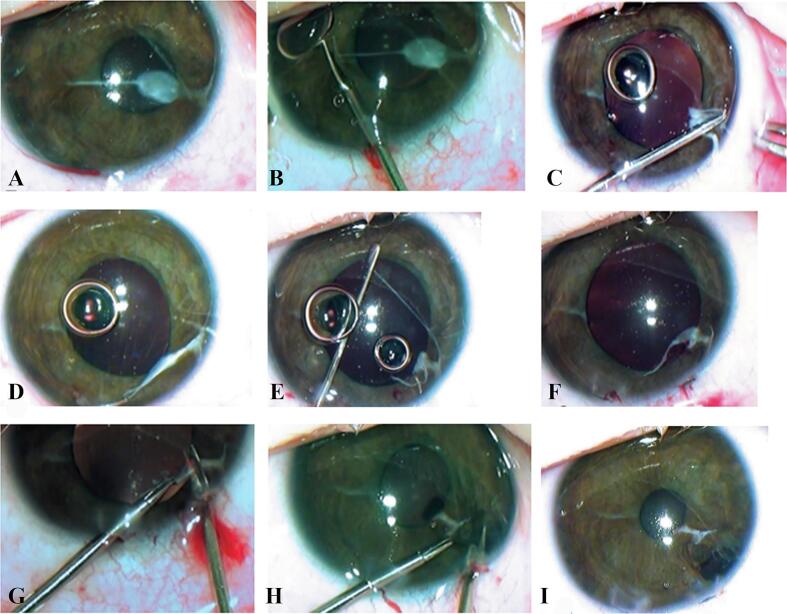
(A–I) Intraoperative photographs of the left eye: A – the pupil is dilated by injecting mydriatic and viscoelastic into the anterior chamber; the pupil dilated unevenly to 4 mm, more superiorly, while the PPM strands, fixed to the transparent lens anterior capsule, did not relocate. B – microscissors were used to dissect the dense PPM strand in the iris tissue at 9 o'clock; C – the dense PPM was gently removed from the anterior lens capsule through circular peeling with vitreous forceps. The anterior capsule of the lens was preserved intact; D – when the membrane is pulled up, the pupil margin can be seen at 4 o'clock and the membrane remains to be fixed in the iris and in the anterior chamber angle; E – blunt removal of anterior embryotoxon from the anterior chamber angle with a spatula; F – PPM retains a tight attachment to the pupil, iris, and anterior chamber angle, together with the removed embryotoxon; G – PPM is cut off the pupillary margin of the iris; H - peripheral iridotomy in the area of angular PPM attachment; small hemorrhages are visible at the site of membrane cutting from the pupil and in the area of iridotomy; I – the appearance of the left eye following the partial removal of the pupillary-iris-lenticular PPM and pupil constriction using myotics. The remnants of the membrane can be seen as white strands tightly fused to the iris at 4 and 9 o'clock. The pupil is round, free, and centrally positioned. The lens is transparent. The peripheral coloboma is located at 5 o'clock.

Limbal paracentesis was performed at 7 o'clock. Following the introduction of mydriatic into the anterior chamber, the pupil exhibited an uneven dilation to 4 mm, predominantly in the superior region. The PPM strands, which were secured to the dense lenticular portion of the PPM on the anterior capsule of the lens, were observed to be stretched but did not displace (Fig. 2A). Following the injection of viscoelastic, the dense strand of the pupillary membrane, which had been tightening the pupil at 9 o'clock, where it exited the iris stroma, was cut with microscissors (Fig. 2B). The dense PPM was separated and removed from the lens surface by gentle circular peeling with vitreous forceps. The anterior capsule of the lens remained intact (Fig. 2C). Using microforceps and a spatula, the lenticular portion of the PPM and the anterior embryotoxon, which it was bound to, were removed throughout the entire anterior chamber angle (Fig. 2D, E, F).

Additional paracentesis was performed at 5 o'clock, through which the PPM and embryotoxon were excised from the pupillary margin and removed from the eye (Fig. 2G). Nevertheless, the pupil continued to be decentered, as a portion of the PPM remained within the iris, subsequently pulling its stroma along with the pupil toward the limbus at 4–5 o'clock. To center the pupil, a peripheral iridotomy was performed at 5 o'clock (Fig. 2H) due to the dense PPM being intertwined with the iris stroma, which made removal impossible. The pupil was narrowed by injecting myotics into the anterior chamber, which allowed it to achieve the correct central position. The iridotomy performed at 5 o'clock resulted in a peripheral coloboma, enabling the displacement of iris tissue along with the PPM located in it (Fig. 2I).

As a result, the proper shape, size, and position of the pupil were fully restored by partially removing the PPM and dissecting the portion of PPM that was tightly woven into the iris stroma. At the same time, the lens's transparency remained intact since the anterior capsule beneath the removed membrane remained clear.

The unremoved portion of the PPM, fused to the iris, remained as two strands at 4 and 9 o'clock radially to the pupil (Fig. 2I).

## 3 Results

The postoperative period was without complications. The child received anti-inflammatory and hemostatic therapy, including systemic intravenous administration of antibacterial drugs and etamsylate, and topical instillation of antiseptic and antibacterial agent in combination with corticosteroid; pupil massage and corneal moisturizing with corneegel were also performed. At hospital discharge, the left eye exhibited a transparent cornea, uniform anterior chamber, transparent aqueous humor, and round pupil, which could dilate and constrict without restrictions, and the anterior capsule and lens remained transparent. VA in the left eye increased after surgery and was 0.13 Teller's cards (0.9 logMAR). The IOP level fluctuated within the normal range and was 18.0–20.0 mm Hg. It was recommended to start training the visual functions of the left eye while patching the right eye.

In the long-term period (12 months), the treated eye exhibited consistent optical and cosmetic outcomes. Visual acuity improved to 0.2 Teller's cards (0.7 logMAR). The IOP remained within the normal range. The dynamics of axial length growth in the treated eye began to align to that of the healthy paired eye. A test with mydriatics showed that the pupil dilated without restriction. It was recommended to continue patching the healthy eye and monitor IOP and lens status.

## 4 Discussion

Corectopia and discoria are distinct pathological abnormalities of pupil formation in the iris; however, they can occur together if the pathological process of iris embryogenesis involves the PPM, which is formed from the tunica vasculosa lentis (lens vascular bag) when reverse development is impaired. It is the PPMs that are observed in corectopia and discoria. They lead to the unification of these signs into one pathological process.

Duke-Elder [4] considered isolated corectopia to be a bilateral disease, but all cases of ITC found in the available literature confirm that this pathology is unilateral.

When visual acuity is high, ITC does not require surgical treatment, allowing for conservative management and follow-up for patients. However, if there's an obstruction in the visual axis that risks the amblyopia development, conservative measures alone are insufficient, and a choice between laser or surgical intervention must be considered.

Single cases of surgical treatment for ITC are described in the literature.

Kumar et al. have reported a case of a boy with unilateral ITC with an oval oblique pupil and a fibrous strand extending from the pupil margin to the cornea. Surgical treatment included dissection of the fibrous strand with scissors, and the central pupil was created by several small incisions in the iris sphincter. According to the authors, pupil displacement can progress during the first months of life, potentially due to a further reduction of the fibrous strand. This is why children with ITC who are at risk of the amblyopia development should undergo laser or surgical treatment [1]. In our case, the restoration of the shape, size, and central position of the pupil was achievable due to the partial removal of the iris-pupillary-lenticular PPM and dissection of the PPM portion that was tightly woven into the iris stroma. This procedure allowed for the restoration of the transparency of the visual axis while preserving the lens's clarity, thereby preventing the amblyopia development.

Atkinson et al. have reported on 4 children with unilateral ITC, which was also caused by a fibrous structure that tethered the iris pupillary margin to the peripheral cornea. Three of 4 children demonstrated progression of the corectopia so two who developed shallow anterior chambers were treated surgically, one with an Nd:YAG laser. The fourth patient was treated with medical mydriasis. Authors recommend medical or surgical intervention for corectopia when the pupillary aperture is displaced peripheral to the central visual axis or when the position of the iris threatens angle structures [2].

Based on our clinical experience, which includes the observation of a notably large number of various PPM manifestations (38 cases), the primary PPM complication in the anterior part of the globe (44.76 %) was the formation of an abnormal shape and size of the pupil [5]. Furthermore, in our case involving pupil formation in congenital acorea, a dense white membrane was observed beneath the iris, which we also considered as a PPM [6]. Therefore, based on our experience in the above cases, the fibrous structure described by Kumar [1] and Atkinson [2], which caused the development of ITC, is nothing more than PPM.

In our case, there was a potential risk of additional displacement of structures to the anterior chamber angle, as the pupil was constricted by the PPM, which was transforming into the anterior embryotoxon. However, the indication for surgical intervention was the presence of pupillary obscuration by the lenticular portion of the PPM, which resulted in a sharp decrease in visual acuity to light perception.

Griener & Lambert have reported the successful treatment of tractional corectopia due to an anterior membrane strand in a child treated with Nd:YAG laser [7].

Robb has described 7 infants who had ITC with PPM. There were 5 various patterns of microcoria pupil displacement in different meridians, depending on the location of the PPM. Six of the 7 patients underwent sphincterotomy. PPM remained in place. The lenses in the area of the newly created pupils were clear. In our opinion, the main limitation of this operation was the loss of the pupil's original shape. Furthermore, the sphincter's capacity to symmetrically constrict and dilate was compromised due to its transection [8].

Brockmann et al. have presented a clinical case of a 2-month-old boy with progressive pupil ovalization, corectopia and ballooning of the thinned superior iris tissue, which caused obstruction of the visual axis. Due to the threat of deprivational amblyopia, sectoral pupilloplasty and sphincterotomies were performed, restoring the shape of the pupil [9].

Ekonomidis et al. have described a case of clinically unilateral non-progressive congenital pupil ectopia in a 37-year-old female who underwent surgical treatment for esthetic reasons. Sphincterotomy was performed with scissors after the preliminary viscoelastic injection, and the pupillary margin of the iris was removed to create a centered round pupil [10].

We found no publications in the available sources that describe the surgical removal of anterior embryotoxon in children, which is due to the fact that such a congenital developmental anomaly usually does not require surgical treatment.

In our clinical case, we were able to perform surgical removal the anterior embryotoxon from the entire anterior chamber angle because it was a continuation of the iris-pupil-lenticular PPM, which caused deformation and decentration of the pupil and obscured the visual axis. Two conclusions can be drawn in this regard:

1) Anterior embryotoxon originates similarly to PPM, as it results from an embryogenesis defect, specifically a disruption in the reverse development of the tunica vasculosa lentis, which is recognised as the cause of PPM.
2) The embryotoxon was removed without the use of cutting tools by delicately separating it from the corneal periphery with collet forceps and a spatula. This removal was without complications for the cornea and anterior chamber angle and did not increase IOP during the postoperative or long-term follow-up period.

## 5 Conclusion

We present a clinical case of congenital ITC complicated by iris-pupil-lenticular PPM, which was fused to the anterior embryotoxon, providing grounds to consider the anterior embryotoxon as a derivative of the tunica vasculosa lentis, as well as the PPM itself.

The delicate microinvasive surgical intervention aimed to restore the shape, position, and size of the pupil. This included the partial removal of the PPM and dissection of the iris in the necessary meridian at the periphery, where a dense PPM strand remained within the iris stroma and was pulling on the pupil. This allowed for the reconstruction of the pupil's normal anatomical position and functions by preserving its sphincter and an intact lens, which, in general, contributed to the rapid restoration of visual acuity and the cosmetic appearance of the eye with ITC.

The position and shape of the pupil, which can dilate and constrict, as well as the preservation of an intact transparent lens with its own accommodative capacity, are both important for restoring the child's vision. Together, these factors allow for a steady increase in visual acuity and full visual rehabilitation of the child.

For the first time, we describe the surgical removal of anterior embryotoxon, which was fused with the structures of the anterior chamber angle. This procedure did not cause complications in the cornea, iris, or anterior chamber angle, nor did it affect the initial normal IOP.

## CRediT authorship contribution statement

BN: Concept, formal analysis, research, methodology, writing, reviewing and editing.
DO: Data curation, research, writing, reviewing and editing.
RT: Formal analysis, data curation, research, writing, reviewing and editing.

All authors have read and approved the final manuscript.

## Consent

Written informed consent was obtained from the patient's parents for publication and any accompanying images. A copy of the written consent is available for review by the Editor-in-Chief of this journal on request.

## Ethical approval

All procedures were performed in accordance with the tenets of the Declaration of Helsinki and approved by the Ethics Committee.

## Guarantor

Nadiia Bobrova.

## Research registration number

N/A.

## Funding

No funding or grant support.

## Declaration of competing interest

None.
